# Barriers to the Utilization of Research and Implementation of Evidence-Based Practice Among Nurses in Sabah, Malaysia: A Cross-Sectional Study

**DOI:** 10.3390/nursrep15070258

**Published:** 2025-07-16

**Authors:** Nadirah Sulaiman, Peter Seah Keng Tok, Juhanah Gimbo, Ammar Rafidah Saptu, Phylis Bridget Philip, Yau Kim Yain, Lilyiana Pengui, Drina Dalie, Norfairuziana Tinggal

**Affiliations:** 1 Clinical Research Centre, Hospital Queen Elizabeth, Kota Kinabalu 88586, Malaysia; juhanah@moh.gov.my; 2 Institute for Clinical Research, National Institutes of Health, Shah Alam 40170, Malaysia; petertok@moh.gov.my; 3 Sabah State Health Department, Kota Kinabalu 88590, Malaysia; abby_rafidah@moh.gov.my (A.R.S.); phyllis@moh.gov.my (P.B.P.); 4 Hospital Duchess of Kent, Sandakan 90000, Malaysia; yauky38@hotmail.com; 5 Hospital Tawau, Tawau 91000, Malaysia; lilyianap81@yahoo.com.my; 6 Hospital Universiti Malaysia Sabah, Kota Kinabalu 88400, Malaysia; drina-d@ums.edu.my; 7 Department of Nursing, Hospital Queen Elizabeth, Kota Kinabalu 88586, Malaysia; fairuzianat@gmail.com

**Keywords:** evidence-based nursing, evidence-based practice, nursing, barriers, Malaysia

## Abstract

B**ackground/Objectives:** Evidence-based practice (EBP) has been widely adopted in clinical nursing practice, with nursing education efforts consistently emphasizing its importance in strengthening implementation efforts. Despite these efforts to promote translational research, the level of implementation of evidence-based practice (EBP) in clinical nursing practice remains unsatisfactory. This study aimed to identify specific organizational, individual, and research-related barriers to the utilization of research in clinical practice among nurses in Sabah, Malaysia, to determine factors associated with these perceived barriers and to assess nurses’ awareness and understanding of the implementation of evidence-based practice. **Methods:** A cross-sectional study was conducted in 2019 using the BARRIERS scale, a validated tool that measures perceived barriers to the utilization of research across four domains: organizational barriers, nurses’ research awareness and values, quality of research, and research communication. This study involved nurses from five tertiary hospitals in Sabah, Malaysia. **Results:** A total of 562 nurses participated in the study, with a mean age of 34.3 years (SD = 7.96) and mean duration of clinical practice of 10.0 years (SD = 7.58). While 66.5% of the nurses had heard of EBP, only 7.3% reported understanding it very well. The top three barriers to the utilization of research were ‘the nurse does not feel she/he has enough authority to change patient care procedures’ (35.9%), ‘the nurse does not have time to read research’ (27.8%), and ‘research reports/articles are not published fast enough’ (25.8%). Among the four domains, organizational barriers scored highest (mean = 2.7, SD = 0.72), followed by research communication (mean = 2.6, SD = 0.73). **Conclusions:** The study findings emphasize the challenges nurses encounter in integrating research into clinical practice and highlight the need for ongoing efforts to promote the utilization of evidence-based practice and research among nurses in Sabah, while addressing the identified gaps.

## 1. Introduction

The utilization of research in clinical practice is essential for the advancement of evidence-based nursing; however, nurses often encounter barriers that impede the integration of research findings into patient care. Integrating research findings into daily nursing practice bridges the gap between theoretical knowledge and practical applications, enabling nurses to make informed decisions based on current and reliable evidence. This approach not only enhances the quality of patient care but also promotes cost-effectiveness and efficiency in healthcare delivery [1].

The recognition of barriers to the utilization of research in nursing has evolved in several ways over time, as evidenced by systematic reviews of existing studies [2,3]. While the most commonly reported barriers have remained largely consistent since the early 1990s, primarily focusing on organizational factors such as lack of time, inadequate resources, and insufficient authority, there have been notable changes in how these barriers are studied and understood in different contexts.

In recent years, the geographical focus of studies on barriers to the utilization of research has expanded from primarily Western European countries to include more research in Asia, Africa, and other regions. Interestingly, despite geographical and cultural diversity, nurses across different regions report similar barriers, such as lack of time, support, and resources, as well as difficulties in understanding statistical analyses [4,5]. These barriers are not only persistent over time but also consistent across continents, indicating a commonality in the challenges faced by nursing professionals [2]. Moreover, factors such as insufficient organizational support, lack of nurses’ research skills, and research communication or accessibility issues have been identified as significant predictors of perceived barriers [6,7,8,9].

While many of these barriers to the utilization of research are commonly encountered across countries, context-specific factors also exist. Evidence from a local setting is imperative to explore and better understand the unique and context-specific barriers to facilitating efforts and policies that are responsive to addressing these barriers. While barriers to the utilization of research in nursing practice have been studied extensively worldwide, it is important to distinguish this specific focus from the broader concept of evidence-based practice (EBP). The utilization of research refers specifically to the process of implementing research findings into practice, while EBP encompasses a wider approach that integrates not only research evidence but also clinical expertise and patient values in decision-making.

In the Malaysian context, several studies have examined aspects of EBP among healthcare practitioners. Lai et al. investigated knowledge and perceptions of EBP among various healthcare professionals, including nurses, identifying some general barriers to the implementation of evidence [10]. Sivasangari et al. assessed the implementation of EBP among nurses in peninsular Malaysia, providing insights into adoption patterns and general challenges [11]. However, a comprehensive examination of the existing literature reveals a gap regarding the specific barriers to the utilization of research—as distinct from the general implementation of EBP—among Malaysian nurses nationally.

This gap is particularly pronounced when considering regional variations within Malaysia. The healthcare landscape in Sabah presents unique challenges compared to peninsular Malaysia, including the geographical isolation of many healthcare facilities, cultural and linguistic diversity affecting the dissemination of knowledge, and different resource allocation patterns. These regional differences may significantly impact how nurses experience and navigate barriers to implementing research findings in their practice. While understanding the general implementation of EBP is valuable, identifying the specific barriers to the utilization of research in this distinct regional context is necessary to develop targeted interventions that address Sabah’s unique healthcare environment.

This study sought to identify specific organizational, individual, and research-related barriers to the utilization of research in clinical practice among nurses in Sabah, Malaysia. It also aimed to determine the factors associated with these perceived barriers and to evaluate nurses’ awareness and understanding of the implementation of evidence-based practice.

## 2. Materials and Methods

### 2.1. Study Design and Settings

This cross-sectional study was conducted between July and December 2019 among nurses from five main tertiary-level hospitals in Sabah, Malaysia. These hospitals were selected, as they serve the majority of the state’s population and employ the largest number of nurses with varying levels of experience, as junior nurses are often placed in these centers for early career training.

### 2.2. Study Population and Sampling

Eligible and practicing nurses from each hospital were randomly sampled and invited to participate in the study. The recruited nurses were required to have at least one year of clinical experience to ensure that they had undergone mentorship programs and had sufficient clinical experience and exposure to identify barriers that they perceived as important to evidence-based practice.

The minimum sample size required for the study was calculated using a sample size calculator to estimate the mean [12]. In a previous study by Shifaza et al. that utilized the BARRIERS questionnaire to assess nurses’ perceptions of barriers and facilitators to EBP implementation in the Maldives, the standard deviation (SD) for various items on the scale ranged up to 1.22 [7]. Using this largest reported SD value (1.22) as a conservative estimate of variability and specifying a desired precision of 0.25 for our mean estimates, the calculated minimum required sample size was 92 participants per hospital. Considering a potential non-response rate of 20%, we aimed to recruit 110 participants (92 participants plus 20%) from each of the five hospitals involved. The total number of participants anticipated for this study was 550, with 110 participants from each of the five hospitals.

A stratified random sampling method was employed to ensure proportionate representation across different hospitals in each district. We used a computerized random number generator to select participants from staff lists provided by nursing management at each facility. This probabilistic sampling approach ensured that nurses across various specialties, experience levels, and shifts were adequately represented.

### 2.3. Inclusion and Exclusion Criteria

To be eligible for inclusion in the study, participants had to meet the following criteria: (1) to be at least 18 years of age, (2) to be registered with the Malaysia Nursing Board, and (3) to have at least one year of clinical practice experience in one of the five participating hospitals. This minimum experience requirement ensured that nurses had undergone mentorship programs and gained sufficient clinical exposure to identify practice-relevant barriers.

Nurses were excluded from participation if they were away from current duty due to an extended period of leave exceeding one month or were unable to provide written informed consent for participation.

### 2.4. Ethical Considerations

This study was registered in the National Medical Research Register (NMRR), and ethical approval to conduct the study was obtained from the Medical Research Ethics Committee (MREC) of the Ministry of Health, Malaysia.

All participants were provided with detailed information about the study’s purpose and procedures through an informed consent form. Written consent was obtained from all participants prior to their enrollment. Anonymity was maintained by using code numbers instead of personal identifiers on all questionnaires. To ensure confidentiality, completed questionnaires were returned in sealed envelopes, and all data were stored securely with access restricted to the research team. Participation was entirely voluntary, and nurses were informed of their right to withdraw at any time without consequences.

### 2.5. Data Collection

This was conducted between July and December 2019 among the nurses. Questionnaires and informed consent forms were distributed by members of the study team to the ward managers in each ward or clinic where the participants were selected. The purpose of the study was explained in detail in the informed consent form, and a copy of the form was provided to each participant. After the participants provided written consent to participate, a questionnaire was administered to them, and they returned the completed questionnaires to the investigator within two weeks.

### 2.6. Research Instrument

This study utilized the BARRIERS questionnaire, a validated tool widely used to assess barriers to the utilization of research in nursing practice [2,13]. The BARRIERS scale consists of 29 items categorized into four subscales: organizational barriers, nurses’ research awareness and values, quality of research, and research communication. Items were rated on a 5-point Likert scale (0 = no opinion, 1 = to no extent, 2 = to a little extent, 3 = to a moderate extent, and 4 = to a great extent). Permission to use the BARRIERS scale was obtained from the original author [14].

While the BARRIERS scale has been widely used across various countries and healthcare settings since its development in 1991, it has also faced criticism in recent literature. Kajermo et al. conducted a systematic review of studies using this scale and questioned its continued relevance, noting that despite decades of identifying similar barriers, little progress has been made in addressing them [3]. Critics argue that the scale focuses predominantly on individual and organizational barriers without sufficiently addressing contextual factors or proposing actionable solutions.

We acknowledge these limitations and have therefore supplemented the BARRIERS scale with additional questions regarding nurses’ perceptions of EBP and context-specific factors relevant to the Malaysian healthcare system. Additionally, we assessed the internal consistency of the BARRIERS scale—both the overall scale and each of the four subscales—in our study samples using Cronbach’s alpha.

### 2.7. Data Analysis

The statistical analysis was performed with IBM SPSS Statistics version 22.0. Descriptive statistical analysis was used to describe the participants’ demographic features, the components of the questionnaire, and the attributes of the subscales of the BARRIERS scale. Mean, standard deviation, and percentage values were used to determine the results. For the BARRIERS scale, a total score was calculated by summing the responses across all 29 items, with each item scored from 1 (to no extent) to 4 (to a great extent). Responses of ‘0’ (no opinion) were treated as missing values and were not included in the calculation of the total score. This approach resulted in a possible score range of 29–116, with higher scores indicating greater perceived barriers to the utilization of research. The mean total score on the BARRIERS scale was compared between groups of variables of interest using an independent *t*-test or One-Way ANOVA. The variables examined included years of experience in the clinical field, highest level of education attained, familiarity with EBP, and perceived understanding of EBP.

For handling missing data, we employed a pairwise deletion approach (also known as available case analysis). Rather than removing entire questionnaires with missing responses, we only excluded specific missing values from relevant analyses. This means that in any particular analysis, only cases with valid data for the specific variables involved in that analysis were included. This approach maximizes the use of the available data by allowing participants with incomplete responses to contribute to the analyses of items they did complete. No imputation methods were used to replace missing values, ensuring that all analyses were based solely on actually reported data.

## 3. Results

A total of 562 nurses were recruited for this study. While the initial target was 550 participants (110 from each of the five hospitals), the number was slightly higher due to additional interest from eligible nurses at some of the participating hospitals. All the nurses who were approached and who met the inclusion criteria agreed to participate in the study.

This study included 562 nurses with varying levels of experience and educational backgrounds. Table 1 presents the detailed demographic characteristics of the participants, including their age, years of clinical practice, qualifications, and educational institutional status. As shown in the table, the majority of participants had completed a diploma-level education and were trained at government institutions.

In the internal consistency assessment, the overall Cronbach’s alpha for the BARRIERS scale was 0.91 in our study samples, indicating excellent internal consistency. The subscale reliability coefficients were as follows: organizational barriers (α = 0.79), nurses’ research awareness and values (α = 0.82), quality of research (α = 0.80), and research communication (α = 0.78). These values indicate good to excellent reliability across all dimensions of the scale in our study population, comparable to those reported in the original validation study and subsequent cross-cultural adaptations [13].

Using the BARRIERS questionnaire (Table A1), an analysis of individual barrier items revealed important patterns across domains. In the organizational barriers domain, lack of authority to change patient care procedures (35.9% reporting ‘to a great extent’) and insufficient time (27.8% for reading research, 25.7% for implementing new ideas) were predominant challenges. Within the research communication domain, the unavailability of research reports (56.4% reporting ‘to a moderate extent’) and unclear practice implications (53.7%) were most problematic. For nurses’ research awareness and values, a lack of capability to evaluate research’s quality (48.2% reporting ‘to a moderate extent’) and isolation from knowledgeable colleagues (41.4%) were key barriers. Regarding research’s quality, concerns about studies not being replicated (50.1% reporting ‘to a moderate extent’) and statistical analyses being difficult to understand (48.9%) were prominent obstacles.

We also investigated possible factors associated with the total mean scores, but none of the variables of interest were found to be significant when the total scores were compared (Table 2). Nurses with ≤14 years of clinical experience had a slightly higher mean total score (75.2, SD = 18.57) than those with ≥15 years of experience (74.4, SD = 22.42), but this difference was not statistically significant (*p* = 0.721). Similarly, no significant differences were found between diploma and bachelor’s degree holders (*p* = 0.145), between those who had or had not heard about EBP (*p* = 0.138), or among the different levels of perceived understanding of EBP (*p* = 0.093). The mean scores for each BARRIERS subscale are summarized in Table 3.

Among the participants, (n = 370, 65.8%) reported having heard of evidence-based practice (EBP), as presented in Table 4. When asked where they learned about EBP, participants most commonly reported their study place (n = 106, 33.7%) or workplace (n = 101, 32.1%), followed by workshops (n = 50, 15.9%), books/journal articles (n = 31, 9.8%), and online sources (n = 23, 7.3%). Approximately half (n = 282, 53.9%) of the respondents considered their knowledge of EBP to be moderate, while only (n = 38, 7.3%) reported understanding EBP very well, and (n = 203, 38.8%) reported not being familiar with EBP.

Approximately (n = 354, 67.0%) of the participants indicated that they had sought information or research evidence to help them in their clinical practice. When asked about their information-seeking practices, the most common approach was conducting general online searches (n = 498, 91.2%), followed by seeking colleagues’ or nurse managers’ opinions (n = 333, 61.0%), reading nursing textbooks and practice guidelines (n = 332, 60.9%), and reading nursing journals/publications (n = 250, 45.8%). Online searches were perceived as both the most important source (n = 171, 39.8%) and the most common approach (n = 298, 68.3%) used by nurses to seek information and research evidence.

## 4. Discussion

Using the BARRIERS questionnaire, we investigated perceived barriers to the utilization of research in clinical practice and found that organizational barriers were the most significant obstacles across all domains (mean score 2.7, SD = 0.72). Among the items perceived to be barriers ‘to a great extent’, three of the top five belonged to the organizational barrier category. In a systematic review, organizational factors were consistently identified as significant obstacles in multiple studies and settings worldwide [2]. Similarly, limitations to authority, insufficient resources and facilities, and the absence of managerial support were among the most commonly reported barriers across geographical regions and practice settings [3].

Our findings revealed that a majority of the nurses surveyed perceived most barriers to the utilization of research as occurring ‘to a moderate extent’. This middle-range perception suggests that while these barriers do not completely prevent the utilization of research, they significantly hinder the implementation of evidence-based practices in clinical settings. The top five barriers endorsed at this level deserve particular attention in the future. Research reports and articles not being readily available (56.4%) emerged as the most commonly endorsed moderate barrier, highlighting significant access problems. This finding corresponds with nurses’ reported information-seeking behavior, where they predominantly rely on general online searches rather than peer-reviewed literature. Similarly, the perception that the implications for practice are not made clear (53.7%) reflects a communication gap between researchers and clinical practitioners that impedes the practical application of research findings.

The concern that research has not been replicated (50.1%) suggests that nurses value reliability in evidence and are hesitant to implement changes based on single studies. This barrier highlights the need for nursing education to emphasize critical appraisal skills to help practitioners evaluate the strength and quality of the available evidence. The difficulty in understanding statistical analyses (48.9%) further supports this need, as quantitative literacy is essential for interpreting the research findings.

Finally, the fact that 48.2% of nurses did not feel capable of evaluating research’s quality (48.2%) reveals a self-efficacy gap that affects their confidence in implementing evidence-based changes. This perception likely stems from limited training in research methodology and critical appraisal during nursing education and professional development courses.

Interestingly, our further analysis found no significant differences in perceived barriers based on years of clinical experience, educational level, or familiarity with EBP among the study participants. This suggests that barriers to the utilization of research in Malaysia are pervasive across different groups of nurses and may be more related to systemic and organizational factors than to individual characteristics. Our results are consistent with those from Jordan, Iran, and international critical care settings, where similar patterns were observed [15,16,17]. However, this observation may be context-specific, as studies in other settings, such as Korea, Cyprus, and China, found that educational background, the understanding of evidence-based practice, and research experience were associated with perceived barriers among nurses [5,9,18].

The findings of this study inform EBP among nurses in Sabah, Malaysia, particularly regarding their perceived barriers to the utilization of research in clinical practice. The majority of the nurses in our study reported having heard about EBP (66.5%), but only 7.3% felt that they understood it very well. This suggests a significant gap between the awareness and in-depth understanding of EBP. Previous studies in various settings have reported similar results. Although nurses in China are aware of EBP concepts, only a small proportion report high confidence in their EBP skills [9]. Similarly, low levels of comprehensive understanding of the utilization of research among nurses in Nepal have been reported, despite reasonable awareness levels [8]. This pattern highlights the need to address the knowledge–practice gap in future training and continuing nursing education efforts.

This study also found that general online searches were the most common practice among nurses seeking information or research evidence (91.2%) and that evidence from these sources was perceived as both common (68.3%) and important (39.8%). While it is encouraging that the majority of nurses reported searching for information or research evidence to support their clinical practice, the heavy reliance on general online searches and colleagues’ opinions (61.0%) is concerning, as the quality and reliability of such evidence may be inconsistent without expert peer review. Comparatively, a smaller proportion of nurses utilized peer-reviewed sources such as nursing journals/publications (45.8%). This finding aligns with a Korean study [5] that found that nurses predominantly relied on informal knowledge sources rather than research literature. This pattern highlights the need to improve access to and training in the use of evidence-based resources.

Our findings have several practical implications for nursing practice, education, and healthcare policy in Malaysia. Based on the identified barriers, this study would like to propose several strategies to enhance the utilization of research. Based on organizational-level strategies, healthcare institutions should implement protected time policies that allocate specific hours within nurses’ schedules for research-related activities. This should be complemented by developing a structured authority framework that empowers nurses to propose and implement evidence-based changes to clinical procedures, giving them greater autonomy in applying research findings. Additionally, creating formalized mentorship programs pairing research-experienced nurses with those less familiar with EBP would help build research capacity and confidence among the nursing workforce, addressing the knowledge and self-efficacy barriers identified in our findings.

In terms of communication and accessibility strategies, addressing the communication barriers identified in our study requires developing a centralized, user-friendly database of relevant nursing research tailored to local clinical contexts, making evidence more readily accessible to practicing nurses. Regular research digest newsletters highlighting key findings relevant to specific nursing specialties would help overcome the barrier of limited time for reading research by providing concise, targeted information. Additionally, simplified research summaries with clear practice applications in multiple languages would address the barriers related to understanding statistical analyses and unclear implications for practice that were prominently reported by participants in our study.

To address the significant knowledge and skill barriers identified in our findings, healthcare institutions should redesign continuing education programs to emphasize practical research skills, including statistical literacy and critical appraisal, which would directly target nurses’ reported difficulties in understanding and evaluating research. Developing tiered evidence-based practice (EBP) competency frameworks, with corresponding training programs for different nursing levels, would create clear pathways for professional development in the utilization of research. Integrating projects applying EBP into nursing performance evaluations would reinforce the importance of research in clinical practice and provide incentives for nurses to develop these skills. Establishing collaborative relationships between academic institutions and clinical settings would bridge the theory–practice gap by creating opportunities for the exchange of knowledge and the practical application of research evidence under expert guidance.

Based on our findings, we recommend that nursing leaders advocate for revised staffing models that account for time needed for the implementation of EBP, addressing one of the most significant barriers identified in this study. Developing position descriptions that explicitly include responsibilities regarding the utilization of research would formalize expectations and elevate the importance of evidence-based practice within the nursing role. Implementing rewards and recognition systems for nurses who successfully integrate research into practice would provide motivation and acknowledge the additional effort required for EBP. Creating policies requiring evidence-based rationales for clinical protocol changes would institutionalize the utilization of research in decision-making processes. Finally, allocating a dedicated budget for research resources and training would demonstrate organizational commitment to evidence-based practice and provide the necessary infrastructure to overcome many of the barriers identified in our study.

Although this study provides valuable insights, it is limited by its cross-sectional nature and reliance on self-reported data. Future research could benefit from longitudinal designs to track changes in perceived barriers over time and interventional studies to test strategies for overcoming these barriers.

## 5. Conclusions

This study provides critical insights into the complex ecosystem of barriers that impede the utilization of research among nurses in Sabah, Malaysia. Our analysis reveals that while awareness of EBP exists, its implementation is hindered by a confluence of organizational constraints, communication challenges, limitations in research literacy, and perceptual barriers that transcend nursing experience levels and educational backgrounds. The persistence of these barriers suggests deep-rooted systemic issues that require coordinated responses across multiple stakeholders.

The predominance of organizational barriers—particularly related to limitations to authority and time constraints—calls for a fundamental reassessment of nursing roles, responsibilities, and workplace structures within Malaysian healthcare settings. Simultaneously, the prevalence of moderate barriers related to access to research, communication, and nurses’ self-efficacy in evaluating evidence highlights the need for enhanced educational approaches and resource allocations.

The translation of an awareness of EBP into its consistent application requires not merely educational interventions but transformational change in organizational cultures, policy frameworks, and the formation of professional identities within nursing. By implementing targeted strategies addressing each barrier domain identified in this study, healthcare leaders can catalyze the integration of research evidence into clinical decision-making, thereby enhancing the quality of care, improving patient outcomes, and advancing the nursing profession in Malaysia.

## Figures and Tables

**Table 1:** Demographic characteristics of nurses who participated in the study (n = 562).

Variables	Categories/Statistics	Frequency (n)	Percentage (%)
Age	Mean (SD)	34.3 (7.96)	-
Years as a clinical nurse	Mean (SD)	10.0 (7.58)	-
Qualification	Certificate Diploma Advanced Diploma Bachelor	16 484 16 46	2.8 86.1 2.8 8.2
Education institutional status	Government Private	439 123	78.1 21.9

**Table 2:** Comparison of total scores among groups of variables of interest.

Variables	n	Mean (SD)	Mean Diff (95% CI) ^a^	*p*-Value ^a^
**Years of clinical experience** ≤14 years 15 years	435 104	75.2 (18.57) 74.4 (22.42)	0.8 (−3.8, 5.5)	0.721 ^b^
**Level of education** Diploma Bachelor	469 42	75.9 (18.09) 80.1 (18.38)	−4.3 (−10.0, 1.5)	0.145
**Have heard about EBP?** Yes No	353 183	76.1 (18.62) 73.5 (20.67)	−2.6 (−6.1, 0.8)	0.138
**Individual’s perceived understanding of EBP** Very good Moderate Not familiar	36 271 198	72.9 (17.21) 76.9 (17.96) 73.1 (21.43)	-	0.093 ^c^

^a^ Independent *t*-test. ^b^ Equal variances not assumed. ^c^ One-Way ANOVA.

**Table 3:** Mean scores for BARRIERS subscales.

Subscale	Mean (SD)	95% CI
Organizational barriers	2.7 (0.72)	2.6, 2.7
Research communication	2.6 (0.73)	2.5, 2.6
Nurses’ research awareness and values	2.6 (0.76)	2.5, 2.6
Quality of research	2.5 (0.79)	2.4, 2.6

**Table 4:** Evidence-based practice (EBP) awareness and understanding among respondents.

Item	Categories	Frequency	Percentage
**Have you heard about EBP?** n = 556	Yes	370	66.5
**Where was it is mentioned?** n = 315	Workshop Study place Workplace Book/Journal article Online Not remember	50 106 101 31 23 4	15.9 33.7 32.1 9.8 7.3 1.3
**How well do you think you understand EBP?** n = 523	Very well Moderately Not familiar	38 282 203	7.3 53.9 38.8
**Do you search for information** **or research evidence to help** **you in your clinical practice?** n = 528	Yes	354	67.0
**Where do you look for** **information or research** **evidence?** *Participant can answer more than one*	Colleagues’ or nurse managers’ opinions	333	61.0
	Search online information (general search)	498	91.2
	Read specific nursing journals/ publications	250	45.8
	Read nursing textbooks and practice guidelines	332	60.9
**The most IMPORTANT** **information source** n = 430	Search online Textbooks and practices guidelines Journals/publications Colleagues’ or nurse managers’ opinions Others	171 109 79 66 5	39.8 25.3 18.4 15.3 0.5
**The most COMMON source used** n = 436	Search online Textbooks and practices guidelines Colleagues’ or nurse managers’ opinions Journals/publications Others	298 64 48 22 4	68.3 14.7 11.0 5.0 0.7

## Data Availability

The data and materials used in this study are not publicly available but can be provided upon reasonable request from the corresponding author: Nadirah Sulaiman, nadirahsulaiman@moh.gov.my.

## Public Involvement Statement

No public involvement in any aspect of this research.

## Guidelines and Standards Statement

This manuscript was drafted in line with STROBE (The Strengthening the Reporting of Observational Studies in Epidemiology (STROBE) Statement: guidelines for reporting observational studies) for cross-sectional research.

## Appendix A

**Table A1:** Evidence-based practice (EBP) among the respondents.

Subscale	Item	This Is a Barrier									
		n (%)									
		To No Extent		To a Little Extent		To a Moderate Extent		To a Great Extent		No Opinion	
RC	Research reports/articles are not readily available	26	(4.6)	112	(19.9)	317	(56.4)	74	(13.2)	33	(5.9)
RC	Implications for practice are not made clear	27	(4.8)	128	(22.8)	302	(53.7)	76	(13.5)	29	(5.2)
RC	Statistical analyses are not understandable	33	(5.9)	132	(23.7)	273	(48.9)	97	(17.4)	23	(4.1)
RC	The research is not relevant to the nurse’s practice	81	(14.5)	149	(26.6)	211	(37.7)	69	(12.3)	50	(8.9)
NRAV	The nurse is unaware of the research	45	(8.0)	112	(19.9)	235	(41.8)	136	(24.2)	34	(6.0)
OB	The facilities are inadequate for implementation	29	(5.2)	116	(20.6)	257	(45.7)	129	(23.0)	31	(5.5)
OB	The nurse does not have time to read research	30	(5.3)	132	(23.5)	221	(39.3)	156	(27.8)	23	(4.1)
QR	The research has not been replicated	27	(4.8)	115	(20.5)	281	(50.1)	92	(16.4)	46	(8.2)
NRAV	The nurse feels the benefits of changing the practice will be minimal	32	(5.7)	104	(18.5)	260	(46.3)	107	(19.1)	58	(10.3)
QR	The nurse is uncertain whether to believe the results of the research	32	(5.7)	121	(21.6)	253	(45.2)	88	(15.7)	66	(11.8)
QR	The research has methodological inadequacies	27	(4.8)	145	(25.8)	236	(42.0)	91	(16.2)	62	(11.0)
RC	The relevant literature is not compiled in one place	24	(4.3)	104	(18.5)	259	(46.2)	126	(22.4)	48	(8.5)
OB	The nurse does not feel she/he has enough authority to change patient care procedures	29	(5.2)	75	(13.3)	216	(38.4)	202	(35.9)	40	(7.1)
OB	The nurse feels the results are not generalizable to their own setting	25	(4.4)	118	(21.0)	242	(43.1)	141	(25.1)	36	(6.4)
NRAV	The nurse is isolated from knowledgeable colleagues with whom to discuss the research	40	(7.1)	135	(24.0)	232	(41.4)	110	(19.6)	44	(7.8)
NRAV	The nurse sees little benefit for self	35	(6.2)	145	(25.8)	245	(43.6)	88	(15.7)	49	(8.7)
QR	Research reports/articles are not published fast enough	29	(5.2)	101	(18.0)	236	(42.1)	145	(25.8)	50	(8.9)
OB	Physicians will not cooperate with implementation	37	(6.6)	128	(22.8)	226	(40.3)	111	(19.8)	59	(10.5)
OB	Administration will not allow implementation	34	(6.1)	141	(25.2)	235	(42.0)	76	(13.6)	74	(13.2)
NRAV	The nurse does not see the value of research for practice	47	(8.4)	147	(26.2)	232	(41.3)	89	(15.8)	47	(8.4)
NRAV	There is not a documented need to change practice	33	(5.9)	110	(19.6)	257	(45.7)	105	(18.7)	57	(10.1)
QR	The conclusions drawn from the research are not justified	29	(5.2)	148	(26.3)	239	(42.5)	63	(11.2)	83	(14.8)
QR	The literature reports conflicting results	35	(6.3)	130	(23.2)	235	(42.0)	91	(16.3)	69	(12.3)
RC	The research is not reported clearly and readably	29	(5.2)	145	(25.8)	221	(39.4)	107	(19.1)	59	(10.5)
OB	Other staff are not supportive of implementation	47	(8.4)	94	(16.7)	229	(40.7)	134	(23.8)	58	(10.3)
NRAV	The nurse is unwilling to change/try new ideas	74	(13.2)	112	(20.0)	215	(38.3)	113	(20.1)	47	(8.4)
QR	The amount of research information is overwhelming	32	(5.7)	130	(23.2)	256	(45.6)	84	(15.0)	59	(10.5)
NRAV	The nurse does not feel capable of evaluating the quality of the research	30	(5.3)	110	(19.6)	271	(48.2)	104	(18.5)	46	(8.2)
OB	There is insufficient time on the job to implement new ideas	34	(6.1)	103	(18.4)	251	(44.8)	144	(25.7)	28	(5.0)

NRAV = nurses’ research awareness and values, OB = organizational barriers, QR = quality of research, RC = research communication.
